# Dry Eye Disease: An Update on Changing Perspectives on Causes, Diagnosis, and Management

**DOI:** 10.7759/cureus.59985

**Published:** 2024-05-09

**Authors:** Nikita Dash, Deepak Choudhury

**Affiliations:** 1 Ophthalmology, Sir Ganga Ram Hospital, New Delhi, IND; 2 Ophthalmology, Maharaja Krishna Chandra Gajapati (MKCG) Medical College, Berhampur, IND

**Keywords:** clinical update, ocular surface, meibomian gland disease, tear film, dry eye

## Abstract

Dry eye disease is a common clinical problem encountered by ophthalmologists worldwide. Interest in this entity has increased in recent years due to the consequences it has on the ocular surface after any surface procedure. With changing times, several new factors have come to light that can influence this disease. The effect of the COVID-19 pandemic has also been greatly felt, with a range of causes, starting from increased screen work to inflammatory processes, exacerbating the condition in many. With changes in the concepts of the etiopathogenesis of the disease, a paradigm shift has taken place in the approaches to treatment. More researchers are in favor of a new tear film-oriented approach that tries to localize the disease to a single component in the tear film. Innovation of newer techniques for the treatment of meibomian gland disease has also made its foray into clinical ophthalmology. Newer drug formulations and molecules are underway to better treat the inflammatory component of the disease. Many other receptors and targets for the treatment of dry eyes are being researched. This review hopes to provide a succinct, narrative summary of the relevant research on dry eye disease to date to increase awareness about the nature and future course of this disease and its management.

## Introduction and background

Dry eye disease (DED) is commonly encountered by ophthalmologists worldwide. In 2017, the Tear Film and Ocular Surface Society International Dry Eye Workshop II (TFOS DEWS II) defined dry eye as “a multi- factorial disease of the ocular surface characterized by a loss of homeostasis of the tear film, and accompanied by ocular symptoms, in which tear-film instability and hyperosmolarity, ocular surface inflammation and damage, and neurosensory abnormalities play etiological roles” [1]. While there has been extensive discussion about the etiopathogenesis of DED, recent consensus in Japan and Asia has attributed tear film instability as the core mechanism behind the disease. The concept of clarifying each cause by focusing on every layer of the tear film is referred to as a tear film-oriented diagnosis [2]. Treatment based on such a diagnosis is referred to as tear film-oriented therapy [3,4]. Recent research has changed the understanding of DED; thus, this review attempts to shed light on newer concepts related to its risk factors and diagnosis as well as changing perspectives on the management of DED.

## Review

Risk factors and their role in disease development

Tear film hyperosmolarity and subsequent inflammatory changes remain the core mechanisms of DED; thus, factors affecting these play an important role in the development of the disease [1]. A recent literature review confirmed the role of increased tear film osmolarity in the pathogenesis of DED and outlined various causes behind it [5].

Environment and Its Contribution

Although the role of environmental factors (e.g. humidity) in DED is often debated, exposure to certain pollutants is known to exacerbate the disease [5,6]. Pollutants such as nitrogen dioxide (NO_2_), sulfur dioxide (SO_2_), ozone (O_3_), and particulate matter (PM) can cause ocular surface symptoms [6]. Both outdoor and indoor pollutants have been found to be associated with the symptoms of DED. The effect of reactive gases on DED has not been studied in detail [7].

In general, high outdoor humidity has been shown to be protective against dry eye symptoms [8]. However, exposure to sunlight, windy conditions, and high altitude can negatively impact the ocular surface [9,10].

Various ocular conditions have been known to cause or aggravate dry eye due to their effect on tear film osmolarity. Sjogren’s syndrome is an autoimmune disease that is largely associated with aqueous-deficient dry eye. The involvement of the lacrimal gland and underlying meibomian gland dysfunction (MGD) play major roles in its pathogenesis. Increased inflammatory markers, such as interleukins and changes in tear film glycoproteins, have also been noted [11-13].

MGD is a type of posterior blepharitis that results in unbalanced lipid secretion, which leads to tear film instability and evaporative dry eye. Many factors, including age, sex, microbial infections, parasitic infestations, and topical medications, are known to influence MGD [14,15]. Ocular surface disorders, such as pterygium and graft-versus-host disease (GVHD), can cause changes in tear composition. A recent proteomic analysis found higher levels of increased expression of keratin proteins in pterygium and GVHD tears, which may be related to increased epithelial keratinization. Similarly, tears from keratoconus-affected eyes showed increased expression of proteins related to immune responses [16]. Pseudoexfoliation syndrome can alter goblet cell activity and mucin production, leading to increased tear osmolarity [17].

Systemic Diseases and Dry Eye

Diabetes is commonly associated with DED, and the prevalence of DED in diabetics may be underestimated. Pathogenesis can be attributed to several factors. Injury to the lacrimal gland, its blood vessels, and/or the corneal nerve following prolonged hyperglycemia can lead to a decrease in aqueous production [18,19], resulting in increased tear osmolarity and blinking abnormalities. The severity of dry eye is often related to the duration of diabetes. Studies have also found that dry eye is more prevalent in patients with diabetic retinopathy and that the severity of DED has a positive correlation with retinopathy [20-22].

Thyroid dysfunction is another cause of dry eye. A mixed mechanism of evaporation and aqueous deficiency has been proposed. Palpebral fissure widening, eyelid retraction, and incomplete blinking lead to inadequate tear distribution and excess tear evaporation [23]. In addition, a wider palpebral fissure results in a shorter tear film break-up time (TBUT), leading to an unstable tear film [24]. Autoimmune mechanisms cause decreased tear production. Autoantibodies against thyroid-stimulating hormone receptors bind to similar receptors on the lacrimal gland, leading to its impairment and aqueous deficiency [25].

Recent studies have found decreased tear volumes in patients suffering from metabolic syndrome. Metabolic syndrome encompasses a group of risk factors for diabetes, hypertension, hyperlipidemia, and other lifestyle diseases. Increased oxidative stress remains the major underlying mechanism of metabolic syndrome [26,27].

Apart from Sjogren’s syndrome, other connective tissue and immune-mediated diseases such as rheumatoid arthritis, systemic sclerosis, systemic lupus erythematosus, and fibromyalgia can present with dry eye [28,29]. Autonomic dysfunction in patients with Parkinson’s disease can also cause dry eye [30].

Lifestyle and Mental Health

Recent studies have found a significant association between TBUT and a sedentary lifestyle, reinforcing the idea that DED is more prevalent among those who exercise less. Prolonged periods of inactivity can predispose individuals to other risk factors and systemic diseases associated with DED [31,32]. In addition, office workers often spend the majority of their time in front of visual display terminals, which results in sympathetic dominance and decreased tear production [33,34], and prolonged visual tasks can result in incomplete or decreased blinks, thereby causing tear film instability and DED [35].

Several studies have found an association between sleep disorders and DED. It is thought that ocular discomfort and inflammatory processes may lead to pain, causing sleep disruption that aggravates symptoms [36,37]. However, it has yet to be established whether poor sleep leads to DED or vice versa. In addition to decreased sleep duration (less than or equal to five hours/night), symptomatic DED patients are more likely to experience psychological stress and have a history of depressed mood [37]. Galor et al. proposed a mechanism of central sensitization in patients with depression and anxiety that may affect how pain is perceived [37]; these patients may react more sensitively to ocular sensations as compared to the control group [38,39]. Subjective happiness has also been found to be positively correlated with dry eye symptoms [40].

Ocular Surgeries

Cataract surgeries have long been considered to aggravate dry eye symptoms. It is believed that corneal incisions made during cataract surgery can release inflammatory mediators (HLA-DR and CD3), similar to the inflammatory process in a dry eye subject [41]. While phacoemulsification cataract surgery does not induce or exacerbate DED in the general population, it can transect the corneal nerves, which may lead to reduced reflex tearing [42]. Preexisting comorbidities, such as MGD and diabetes, play a major role in increased dry eye symptoms following cataract surgery [42]. Manual small-incision cataract surgery results in more symptoms due to the size of the incision involved, which results in the denervation of a greater part of the cornea [43].

Recently, Ju et al. have demonstrated that femtosecond laser-assisted cataract surgery (FLACS) can lead to tear film instability by changing the corneal curvature prior to surgery, which can damage the limbal stem cells and conjunctival goblet cells [43]. Photorefractive surgeries often lead to iatrogenic corneal nerve damage, changes in corneal shape, and damage to conjunctival goblet cells, all of which cause decreased tear production, impaired wettability, and increased tear osmolarity, thus resulting in dry eye symptoms [44,45]. Although dry eye symptoms have been reported with the implantable collamer lens technique, it is moderate compared to laser vision correction [46]. Small incision lenticule extraction (SMILE) reported better TBUT and Ocular Surface Disease Index (OSDI) scores as compared to laser-assisted in situ keratomileusis (LASIK). Significant differences in neuromediators and proteomics of the tear film have been observed between the two groups even when ocular surface signs are comparable [47,48].

Changes in corneal sensitivity, tear film instability, and goblet cell loss have been attributed to the DED symptoms in strabismus patients post-surgery. Exposure to a larger area of bulbar conjunctiva opposite the side of deviation and distortion of the normal relationship between the lids and the globe leads to microtrauma, which can cause symptoms prior to surgery [49]. A recent study showed an increased concentration of inflammatory tear mediators (IL-6 and TNF-α) in concomitant exotropia, which explains the prevalence of dry eyes and tear film instability in squint patients before surgery [50].

Effect of COVID-19

The COVID-19 pandemic heralded a new era of DED, primarily due to increased screen usage and lifestyle changes. Napoli et al. coined the term “quarantine dry eye” while discussing lifestyle factors such as diet, hydration, sleep deprivation, and other psychological aspects pertaining to restrictions imposed by the pandemic [51]. Besides, the use of screens doubled, leading to increased eye strain and dry eye symptoms [52]. Moreover, the mandatory usage of masks contributed to worsening dry eye symptoms, especially in females and subjects with prior DED [53].

Changes to the ocular surface after a COVID-19 infection can also cause DED. Compared to healthy eyes, in vivo confocal microscopy (IVCM) in COVID-19 survivors has established the loss of small corneal nerve fiber and increased dendritic cell density at a mean of 3.7 months after diagnosis [54]. Moreover, the ocular surface also serves as an entry point for SARS-CoV-2, since angiotensin-converting enzyme 2 and transmembrane protease serine 2 were detected in conjunctiva and cornea, probably upregulated by the inflammatory process [55]. Patients with long COVID may also present with worsening MGD and ocular surface staining scores, with higher viral loads and supplementary oxygen use being major risk factors in them. Bilateral dacryoadenitis, wherein fibrosis and obliteration of lacrimal gland ducts have been observed, has been reported as a post-COVID complication [56]. This may play a role in the pathogenesis of DED in COVID-19 survivors.

Other Causes of DED

While advancing age is a well-known risk factor for DED, sex and hormones also play important roles [57,58]. The recent TFOS DWES II report not only agrees with the fact that females are more affected than males but also establishes that DED is more likely to affect women at a younger age than men. Several anatomical and psychological factors, such as pain perception and mental health, are culpable [58].The report also acknowledges the role of hormones in the development of DED. Androgens are an important regulator of the ocular surface and adnexa, especially the meibomian gland; thus, their deficiency may trigger a non-autoimmune type of DED known as primary lacrimal gland deficiency, with associated MGD [58]. Other hormones, such as thyroid hormones, hormones of hypothalamic pituitary axis, and steroid hormones, have also been shown to influence the ocular surface and adnexa, although a clear mechanism is yet to be established [58].

The use of contact lens and topical medications with preservatives can also prove detrimental to the ocular surface, leading to increased tear osmolarity and subsequent dry eye symptoms [5,59]. Other risk factors include Asian race, allergies, vitamin A deficiency, and essential fatty acid deficiency [60].

Changing concepts in diagnosis and advancements in diagnostics

Traditionally, dry eye has been diagnosed based on clinical findings from Schirmer’s test, TBUT, staining of cornea and conjunctiva, and standardized scoring systems. However, recent research on tear film break-up analysis patterns, tear film osmolarity, and biomarkers has provided deeper insights into the diagnosis of DED.

Tear-Film-Oriented Diagnosis and Fluorescein Break-Up Patterns

The Japanese and Asian dry eye societies consider an unstable tear film at the core of dry eye mechanisms; thus, the concept of tear-film-oriented diagnosis was introduced to clarify the cause by focusing on each layer of the tear film. Yokoi et al. have described fluorescein break-up patterns that not only aid in diagnosis but also detect which tear film layer is responsible for DED. The break up patterns judge the abnormalities in the tear film using a physical theory [3,4]. When there is upward movement of the eyelid during eye opening, capillary suction from the upper tear meniscus sucks the aqueous tears up leading to the spread of aqueous layer on the cornea. However, the surface pressure gradient on the tear-film lipid layer pulls the aqueous tear layer resulting in the formation of the precorneal tear film. The various tear-film break-up patterns are area break, spot break, line break, dimple break, and random break, which are described in Table 1 [3].

**Table 1:** A summary of different patterns of tear-film break-up and their mechanisms as described by Yokoi et al.

Type of break	Description
Area break	Fluorescein break-up is observed during eye opening when aqueous tear volume is severely reduced
Spot break	Characteristic spot-like shape noted immediately after eye opening. This is a result of impaired wettability due to mucin deficiency
Line break	It occurs after eye opening due to drag and suction mechanisms. It is characterized by a linear break in the lower part of the cornea in mild-to-moderate aqueous deficiency
Dimple break	A vertical line-like break in the tear film towards the end of the upward movement of fluorescein-stained tears. It is caused by a mild-to-moderate impairment of wettability due to mucin deficiency
Random break	It is caused by the increased evaporation of tears. This results in the thinning of the aqueous layer that causes a random break after the movement of the fluorescein-stained tear film is complete.

This is a very basic interpretation of DED types based on fluorescein break-up patterns. Yokoi et al. described more complex and modified forms based on physical theory and the forces involved [61]. It is believed that the practical use of such a classification might make it popular in the years to come.

Tear Film Osmolarity and Its Measurements

The osmolarity of the tear film explains homeostasis involving tear production, drainage, absorption, and evaporation. Presently, a threshold of 316 mOsm/L is used to differentiate between mild and moderate/severe dry eye, whereas a threshold of 308 mOsm/L is considered sensitive enough to distinguish normal eyes from those with DED [62]. The tear film osmolarity measurements show great variability in patients with DED. Therefore, an average reading is considered more reliable than a single measurement [63].

There are three primary techniques for the measurement of tear film osmolarity: electrical impedance, vapor pressure, and freezing pressure osmometry [64]. Devices such as the TearLab osmometer and I-Pen® use the electrical impedance technique, which measures tear film osmolarity based on the number of charged particles therein. The Wescor 5520 Model follows the concept of vapor pressure; an advantage is the ability to measure osmolarity in small tear film samples. Freezing pressure osmometers are the gold standard, as they can accurately measure osmolarity in very small tear volumes. However, they are highly operator-dependent and require extensive apparatus, and a single measurement takes about 15 minutes [64].

Concept of Functional Visual Acuity

Dry eye patients usually complain of visualization difficulties despite good visual acuity. This has led to the idea of dynamic measurement of visual acuity in such patients. Goto et al. first proposed the measurement of visual acuity by forced eye opening under topical anesthesia and termed it “functional visual acuity” (FVA) [65]. They concluded that FVA decreased despite good conventional visual acuity. Manual measurements, however, resulted in inter-test variations and drawbacks associated with the timing of measurements [64].

Presently, the FVA measurement system provides a non-invasive alternative to continuous visual acuity monitoring under natural blinks without the use of topical anesthesia. In addition to its use in the diagnosis and screening of DED, it has been of great help in judging the effect of dry eye treatments, as improved FVA has been noticed in those undergoing treatment [66]. However, numerous factors can influence FVA assessment, and future research in this matter is required to explain abnormal FVA measurements [66].

Non-invasive Imaging Modalities

To avoid disturbances caused by fluorescein instillation, lipid layer interferometry, a grid xeroscope, and a tear film stability analysis system have been used to study tear film stability. The tear film stability analysis system uses corneal topography. Goto et al. proposed capturing images immediately and 10 seconds after eye opening to study the changes in surface regularity and asymmetry. Later, analysis using aberrometry was introduced to detect higher-order aberrations in DED [65,66]. Newer instruments, such as the Oculus Keratograph 5M, not only measure TBUT but can also evaluate meibomian glands and measure tear meniscus height (TMH) [67].

Anterior segment-optical coherence tomography (AS-OCT) has also emerged as a valuable tool for measuring TMH and assessing tear volume. A dry eye diagnosis is made with a sensitivity of 67% and a specificity of 81% when 0.3 mm of the inferior AS-OCT meniscus height is the cut-off. Its use in the assessment of TMH following therapeutic procedures has also been described [68,69].

Another instrument that has been developed for the measurement of tears is the strip meniscometry tube. The commercially available Strip meniscometry tube (SMTube, Echo Electricity, Fukushima, Japan) uses a non-woven rayon and pulp material sandwiched between polyethylene material as the absorber. It can measure the retained tear volume in the tear meniscus in five seconds. Its cut-off value for diagnosing dry eye is considered as 4 mm, with 93% sensitivity and 73% specificity. The validity of the measurements has been correlated alongside AS-OCT values [66,70].

While impression cytology has been used to study the cellular morphology of the ocular surface in the past, today, IVCM provides a non-invasive alternative. The histological trademark of DED in a typical conjunctival epithelium is squamous metaplasia, which has been detected by both impression cytology and IVCM without any significant differences. Although a morphological study of goblet cells did not yield consistent results, certain parameters related to MGD that could aid in the diagnosis and study of the disease have been detected with greater sensitivity and specificity with ICVM [66].

Advances in MGD Diagnosis

In addition to meibography and confocal microscopy for MGD diagnosis, recent developments in lipid layer interferometry have further expanded the armamentarium for MGD diagnosis. These include the LipiView® (TearScience Inc., Morrisville, NC, USA) and DR-1α® (Kowa, Nagoya, Japan). LipiView quantifies lipid layer thickness in the lower one-third of cornea, while the DR-1α assesses the lipid layer and evaluates the entire cornea, including the central region and tear film dynamics. In addition to facilitating MGD diagnosis, these devices help assess disease severity and create treatment plans for subjects with MGD [66].

*Tear* *Film Biomarkers*

Because inflammation lies at the root of DED pathogenesis, several studies have attempted to find a suitable biological marker that can be measured and quantified to aid in the diagnosis and/or prognosis of the disease. Proteins such as S100A8, S100A9, lipocalin, and α-1 antitrypsin have been observed in greater quantities in the tears of dry eye patients than those of controls. Increased expression of interleukins, protease inhibitors, and tumor necrosis factor-α has also been detected. It has also been found that matrix metalloproteinase-9 (MMP-9) found in ocular inflammation can cause DED. One study has established that certain chemokine levels are positively correlated with TBUT, basal tear secretion, goblet cell density, tear clearance rate, and keratoepitheliopathy score [64]. However, no consensus on the ideal biomarker has been reached.

Advances in dry eye treatment

Following a tear-film-oriented diagnosis, therapy to treat DED caters to the patient needs and is tear-film-oriented. The therapeutics already in use is outlined in Table 2.

**Table 2:** Treatment options in use for DED. DED: dry eye disease; MGD: meibomian gland dysfunction. Table credit: Author Nikita Dash.

Aqueous layer treatment	Mucin layer treatment	Anti-inflammatory
CMC 0.5% or 1%	Rebamipide	Cyclosporine A 0.05%
Sodium hyaluronate 0.1 or 0.3%	Diquafosol sodium 3%	Autologous serum drops
Diquafosol sodium 3%	-----	Preservative-free corticosteroids
Polyvinyl alcohol	-----	MGD treatment
Punctum plugs	-----	Omega-3 fatty acids

Progress in research and development of new drugs has added to the arsenal against DED.

Advances in Therapeutics Targeting Meibomian Gland

Devices for warm compresses: While conventional warm compresses have been proven to be effective for the treatment of MGD by liquefying meibum and changing lipid layer composition, several modifications have been attempted. Some studies have suggested the addition of menthol to improve TBUT; however, the amount of improvement could not be clearly attributed to menthol. The wetness of warm compresses has also been debated, with Arita et al. theorizing that increased wetness leads to a cooling effect that can undermine the benefits of warm compresses. Other devices, such as MGDRx eye bags, EyeGenie masks, and Blephasteam, have also shown better results compared to conventional towel compresses. Their success has been attributed to their ability to maintain an appropriate warm temperature of the eyelids for a longer duration [71].

Thermal pulsation technique: LipiFlowTM (TearScience Inc., Morrisville, NC, USA) is a device that combines meibomian gland expression with heat in an in-office MGD treatment technique known as vector thermal pulse therapy. The device applies heat to the palpebral conjunctiva of the upper and lower eyelids, while providing pulsatile external pressure. Studies have shown that this device can alleviate symptoms for up to three years [66,71]. The MiBo Thermoflo (MiBo Medical Group, Dallas, TX, USA) also uses the same principle, but ultrasound energy is used for massaging. However, it does not raise the temperature to more than 40°, which is optimum for meibum liquefaction. Randomized trials are awaited in this regard [72,73].

Probing of meibomian gland: A novel technique of meibomian gland probing using a bevelled, 2 mm stainless steel probe into meibomian orifices was first described by Maskin in 2010. Patients reported immediate relief that lasted for four weeks. In 2018, post-probing meibography revealed the lengthening of shortened glands and the partial restoration of faded glands with the possibility of new gland growth [74,75]. Syed and Sutula described a new method using an operating microscope [76]. While the symptoms of MGD have been found to be relieved by both methods, they tend to reappear after an average of 38.2 weeks after initial treatment. Therefore, multiple sessions of probing are necessary for long-term success [71].

Intense pulsed light therapy: It helps to liquefy the meibum for easy expression, retard bacterial and parasitic growth on eyelids, induce thermolysis of telangiectatic vessels around the gland, and stimulate cell activity in meibomian glands that promotes wound healing [71]. Studies have shown that IPL treatment is effective against MGD-associated dry eye of varying severity, although at least three treatment sessions two to four weeks apart are required for desired results. It has recently been approved by the United States Food and Drug Administration for dry eye treatment.

Topical and oral medications: Topical and oral azithromycin formulations have been effectively in use for the treatment of bacterial colonization and inflammatory processes associated with MGD. Research has shown that the differentiation of human meibomian gland epithelial cells (HMGECs) is induced by the cationic amphiphilic nature of azithromycin. Similarly, solithromycin, a newer macrolide, has been found to be more potent in stimulating HMGECs; however, clinical trials are pending. Oral tetracyclines also show anti-inflammatory property owing to their bacteriostatic nature and ability to reduce MMP-9 on the corneal surface [71].Other topical medications that have been studied include Manuka honey, lipid-containing eye drops, and perfluorohexyloctane eye drops, all of which have been shown to be effective as adjunctive therapy for MGD-related dry eye with few local side effects [71]. They act by improving meibum quality, gland expressivity, and alleviating the symptoms of blepharitis [71].

Other devices treating MGD: BlephExTM and TearCare® System (Sight Sciences, Menlo Park, CA, USA) are two recently developed in-office treatment devices that aim to provide symptomatic relief against MGD. The BlephExTM is a handheld device that spins a disposable, single-use medical-grade microsponge to remove debris from lashes and lids and helps in the treatment of blepharitis. Although Connor et al. demonstrated improvement in symptoms four weeks after treatment, a trial is yet to be conducted [77,78]. The TearCare® System (Sight Sciences, Menlo Park, CA, USA) places single-use, flexible iLidTM applicators over each tarsal plate, which deliver constant, regulated heat at 41-45°C over a 12-minute treatment time. This helps in meibum liquefaction and effective expression. A randomized controlled trial is awaited; however, manufacturer-sponsored trials have shown improvement in symptoms [78].

Newer Drugs and Potential Therapeutics

Lifitegrast: In July 2016, lifitegrast 5% became the second FDA-approved topical ocular anti-inflammatory medication for the treatment of DED. It blocks interactions between intercellular adhesion molecule-1 (ICAM-1) and lymphocyte function-associated antigen-1 (LFA-1), which are instrumental in T-cell activation and migration. The results of phase III clinical trials (OPUS 1-3) demonstrated improvement in both signs and symptoms of DED. Improved primary symptom outcome of change from baseline eye dryness score (EDS) within 14 days and effect sustained till study endpoint of 84 days. Further study is needed to determine which patients respond best to lifitegrast and whether concomitant use of lifitegrast with topical corticosteroids or cyclosporine can result in faster symptomatic relief [78,79].

Lacritin: Ocular-specific glycoproteins secreted primarily by acinar cells of the lacrimal gland increase basal tear secretion. Studies have shown that lacritin levels are significantly decreased in patients with Sjogren’s syndrome as compared to healthy controls [80]. The efficacy of lacritin in humans was established in 2021, although dosing and duration of administration have yet to be finalized.

Lubricin: Lubricin (PRG4) is a mucin-like glycoprotein that is expressed by the normal ocular surface and suppressed by inflammatory cytokines. It reduces friction between the cornea and the conjunctiva and eyelid. With regard to symptomatic relief, research has shown that lubricin (150 mcg/mL) significantly outperformed sodium hyaluronate (0.18%) in moderately DEDs [81]. A phase-II trial was completed in the United States by Novartis.

Thymosin ß4: Thymosin ß4 is a G-actin-binding protein that improves epithelial healing and downregulates pro-inflammatory cytokines. Sosne et al. first reported the use of topical thymosin ß4 (RGN-259) for the treatment of DED [82]. In 2018, a head-to-head comparison of RGN-259 versus prescription agents including cyclosporine, lifitegrast, and diquafosol was done in a murine model. The RGN-259 drops performed comparably or better [83]. RGN-259 is currently the subject of a 700-patient phase 3 clinical trial in the United States and has demonstrated safety in the treatment of DED consistent with previous clinical trials [84].

Higher concentration cyclosporine: Eye drops with a higher concentration of cyclosporine (CsA) than Restasis® have been approved in Europe, Japan, and other countries. Phase II/III results, reported by Tauber et al., demonstrate an earlier onset of action in the 0.09% formulation with similar safety and tolerability profiles. A phase III clinical trial is currently underway for SecieraTM (Sun Pharma, Mumbai, India), which has a CsA concentration almost twice that of Restasis® (0.09% vs. 0.05%) [78,85].

Amniotic membrane extract eye drops: Amniotic membrane extract eye drops (AMEED) are proposed to have growth factors, cytokines, and collagens that promote corneal wound healing, inhibit fibroblast activity, and decrease inflammation on and within the ocular surface. A variety of products that are marketed for the treatment of DED are available without FDA regulations, and clinical trials are ongoing for use in patients with GVHD and post-PRK dry eye [86].

New Devices for DED

Thermosensitive collagen plugs: The thermosensitive atelocollagen punctal plug is initially liquid; when inserted from the punctum, it solidifies with the body temperature and the punctum is occluded. In order to maximize the effect of this thermosensitive atelocollagen punctal plug, a pre-heating method has been described where the plug is preheated to 41°C and injected in a somewhat stiffened state [87].

Modified moisture chamber spectacles: Modified moisture chamber spectacles have a cover integrated with the frame of the glasses and a tank for containing water to increase humidity around the eyes. Furthermore, ultrasonic moisture glasses can be used to actively control humidity [88,89].

Scleral lenses: The most well-known scleral lens used to treat ocular surface disease is the BostonSight® PROSE (Prosthetic Replacement of the Ocular Surface Ecosystem) lens (Boston Foundation for Sight, Needham, MA, USA), which can significantly improve dry eye signs and symptoms due to Stevens-Johnson syndrome, chronic GVHD, and even neurotrophic keratopathy following skull base tumor resection. Other innovations include the EyePrintProTM lens, which converts an imprint of the ocular surface into a 3D digital model [78,90].

Tear neurostimulation: TrueTear® stimulates the intranasal nerve endings of the afferent nasolacrimal reflex pathway in the nasal mucosa with small electric currents to increase tear production. Improvements in Schirmer scores were observed after using the device four times a day for 180 days. Common side effects include nasal discomfort, burning, pain, nosebleeds, transient electrical discomfort, nasal congestion, facial pain, and headaches. They are contraindicated in patients with implantable metallic or electronic devices and in those with a hypersensitivity to hydrogel that coats the device probes [91].

Antioxidants and Probiotics

Anthocyanin-rich berries and berry extracts are food supplements that have been used for the treatment of eye diseases based on the findings of animal and human studies. A recent trial concluded that the maqui berry has the maximum effect against reactive oxygen species in the lacrimal gland. Similarly, probiotics like *Enterococcus faecium* WB2000 have reportedly improved dry eye symptoms in human subjects and murine dry eye models [66].

Future Research

The potential for future research remains targeted at anti-inflammatory mechanisms for the treatment of DED. Proposed therapeutic targets include interleukin-20, corneal lymphangiogenesis, the PI3K/AKT signaling pathway, and microRNA-328 [92-95]. Newer drugs, such as IL-1 receptor antagonists, serine protease inhibitors, resolvin analogues, and integrin antagonists, are under research [96]. The therapeutic role of pituitary adenylate cyclase-activating polypeptide is also being explored, and drug delivery systems, such as liposomal rebamipide, are being considered [97,98].

## Conclusions

Despite being a very common clinical problem, several aspects of DED are poorly understood and require further research. The role of environmental conditions needs to be studied in depth for a better understanding of the disease pathophysiology and its significance in inciting the inflammatory processes that result in dry eye. Future research could also include the role of the genetic profile of an individual in the causation of DED. With the advent of artificial intelligence, there is a probability to screen DED using algorithms in the future. With newer imaging modalities and interesting research in the pipeline, it remains to be seen what more can be done for DED patients.
